# Supplementary material for: Limitations of acyclovir and identification of potent HSV antivirals using 3D bioprinted human skin equivalents
Source: Nat Commun. 2025 Oct 16;16:9200. doi: 10.1038/s41467-025-64245-w (PMC12533247; doi:10.1038/s41467-025-64245-w)
Supplement: Supplementary file 1 — Supplementary Information [file 41467_2025_64245_MOESM1_ESM.pdf]

SUPPLEMENTARY INFORMATION

Limitations of acyclovir and identification of potent HSV antivirals using 3D bioprinted human skin equivalents

S. Tori Ellison[1†], Ian Hayman[2†], Kristy Derr[1], Paige Derr[1], Shayne Frebert[1], Zina Itkin[1], Min Shen[1], Anthony Jones[2], Wendy Olson[2], Lawrence Corey[2, 3, 4], Anna Wald[2, 3, 4, 5], Christine Johnston[3, 5], Youyi Fong[4], Marc Ferrer[1*], Jia Zhu[2, 4, 6*]

[1]Department of Preclinical Innovation, National Center for Advancing Translational Sciences, National Institutes of Health, Rockville, Maryland 20850, USA.

[2]Department of Laboratory Medicine and Pathology, University of Washington School of Medicine, Seattle, WA 98195, USA.

[3]Department of Medicine, University of Washington School of Medicine, Seattle, WA 98195, USA.

[4]Vaccine and Infectious Disease Division, Fred Hutchinson Cancer Center, Seattle, WA 98109, USA.

[5]Department of Global Health, University of Washington School of Medicine, Seattle, WA 98195, USA.

[6]Institute of Stem Cell and Regenerative Medicine, University of Washington, Seattle, WA 98195, USA.

† These authors contributed equally to this work.

*Corresponding author emails: marc.ferrer@nih.gov; jiazhu@uw.edu

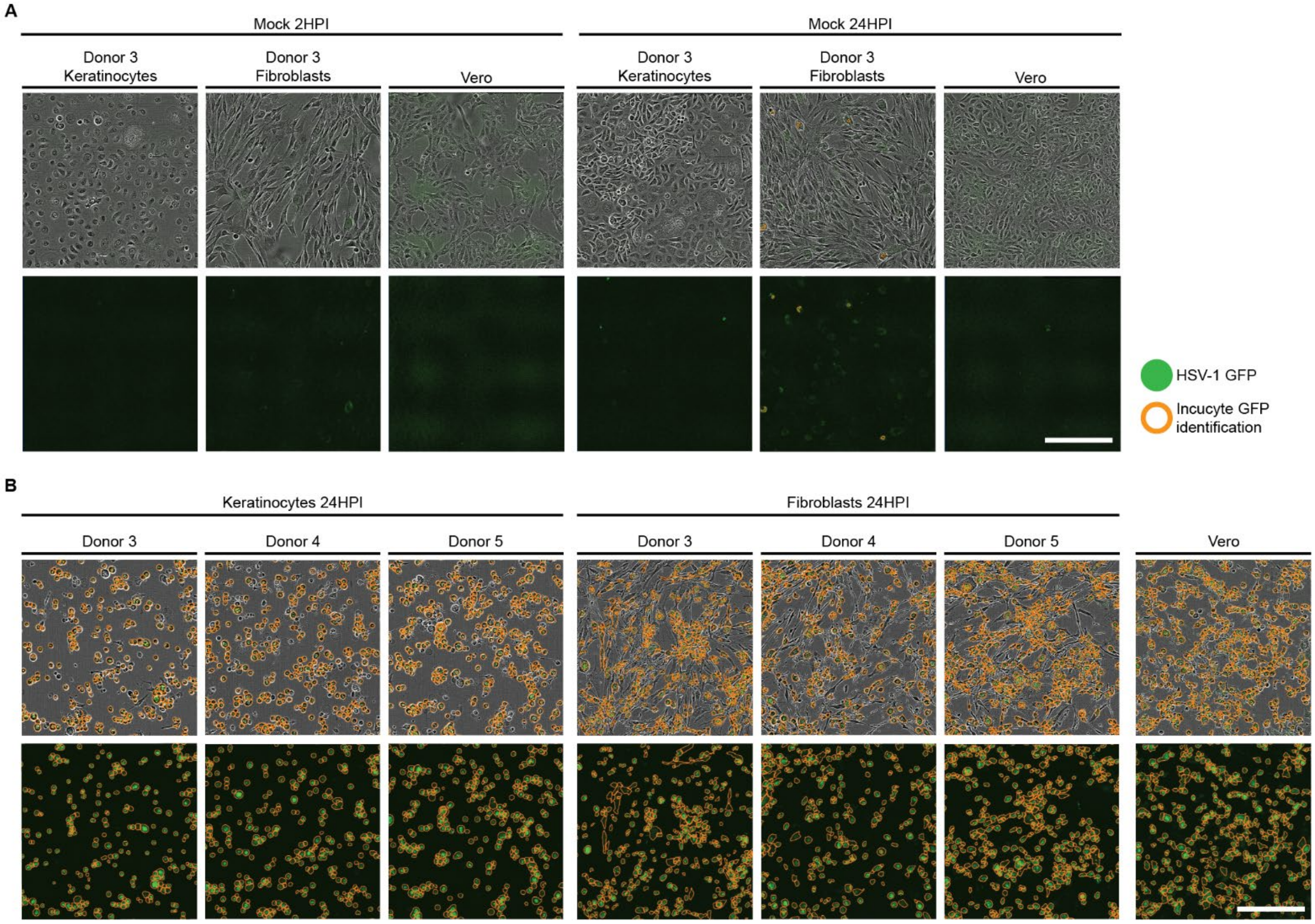

Supplementary Fig. 1. Validation of Incucyte automated identification of GFP fluorescence.
(A) Representative images of mock-infected cell cultures at 2 hours post-infection (HPI) and 24HPI were used to establish background autofluorescence. (B) HSV-1 GFP infected keratinocytes, fibroblasts, and Vero cells at 24HPI were outlined (in orange) to confirm GFP-positive cells identified by the Incucyte software. Scale bar is 250 μm.

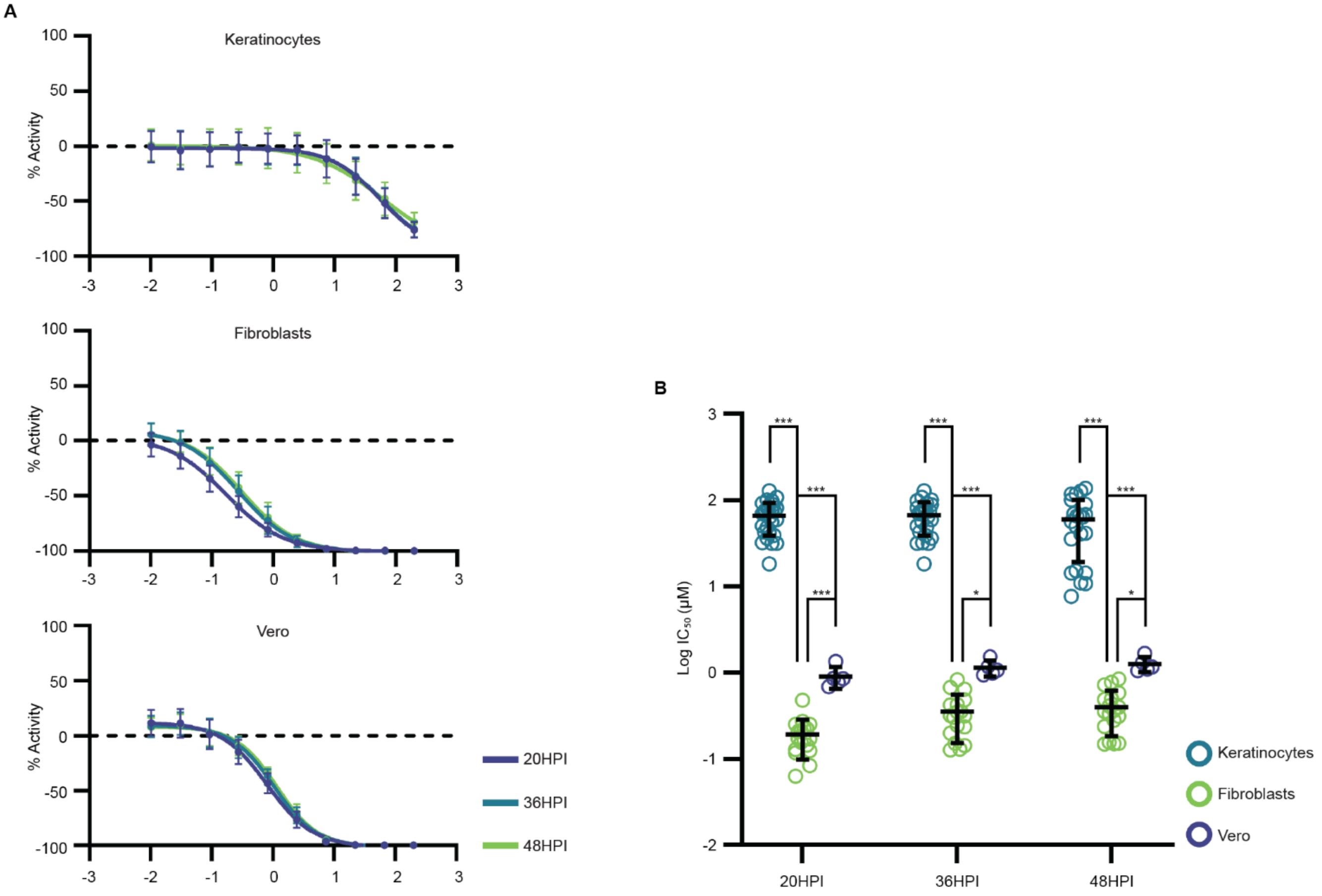

Supplementary Fig. 2. Acyclovir dose response and IC_{50} in Vero cells, keratinocytes, and fibroblasts at 20, 36, and 48 hours post infection

(**A**) Dose response curves for Vero cells, keratinocytes, and fibroblasts at each of the specified times. Cell type is specified in the y-axis of each plot. Average curves represent N = 30, 18, and 5 for keratinocytes, fibroblasts, and Vero cells respectively. Error bars are single standard deviations. **(B)** Calculated IC_{50} values for acyclovir in each cell type at each of the specified times (* $P = 0.013$, *** $P < 0.001$, linear mixed model). Average for each cell type is plotted, individual biological replicates are represented by symbols and error bars represent a single standard deviation. Keratinocytes (six donors and five biological replicates per donor), fibroblasts (six donors and three biological replicates per donor), and Vero cells (five biological replicates) respectively.

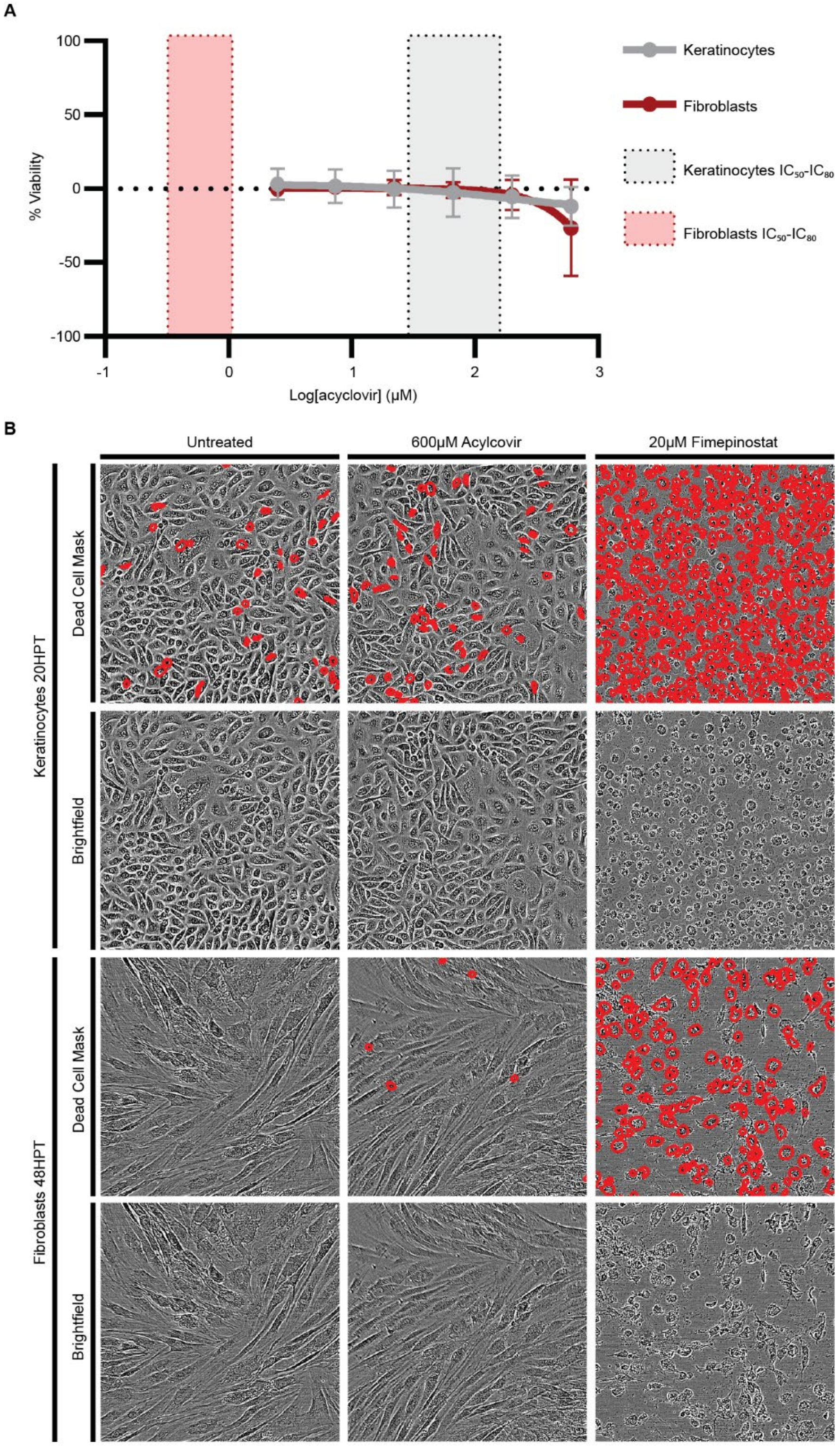

Supplementary Fig. 3. Cytotoxicity of acyclovir in primary keratinocytes and primary fibroblasts. **(A)** Toxicity dose-response curve of acyclovir in primary donor-derived keratinocytes (grey) and fibroblasts (red). Boxes represent the dose range of the IC_{50} through IC_{80} in each cell type. Dose response curves generated using three biological replicates of six keratinocyte donors (N = 18) and two biological replicates of six fibroblast donors (N = 12) **(B)** Representative brightfield images of each cell type following treatment with acyclovir (600 μM) or fimepinostat (20 μM, positive control). Keratinocytes and fibroblasts were treated under the same conditions as described in Fig. 1. Red outlines denote cells identified as non-viable by the Incucyte.

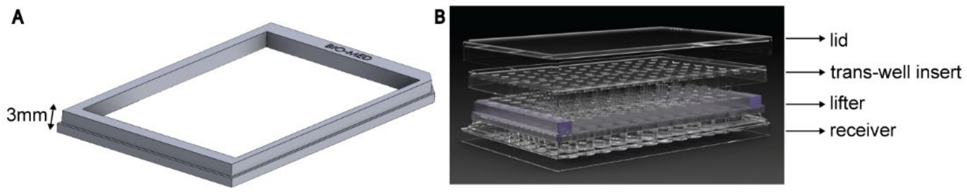

Supplementary Fig. 4. 3D printed lifter design for generating ALI cultures.
(A) Render of 3D printed lifter used to elevate 96-well transwell inserts to generate ALI cultures. **(B)** Depiction of lifter application within a standard 96-well transwell plate. Lifter is made of SBX-compliant material.

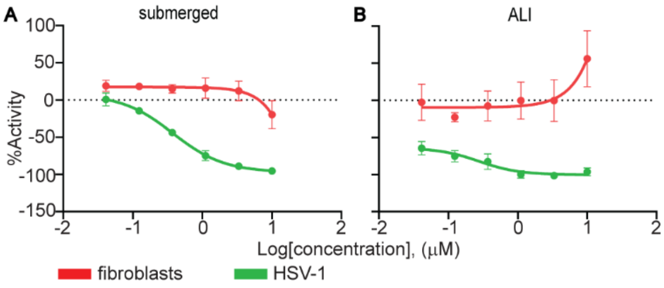

Supplementary Fig. 5. Dose response of acyclovir in submerged and ALI cultures.
(A) Acyclovir dose response (green) in the submerged model and in the **(B)** ALI model. Fibroblast transfected to express tdTomato signal (red) measures cytotoxicity. Average curves (N = 6 biological replicates) are plotted with error bars representing a single standard deviation.

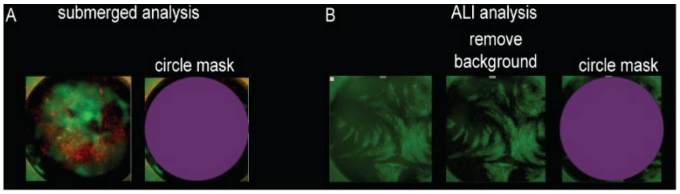

Supplementary Fig. 6. Background fluorescence signal mitigation in 3D bioprinted assay.
(A) In the submerged model, the false fluorescent signal of the plastic well edges is removed by using a circle mask. Only the signal within the circle mask is measured. **(B)** In the ALI model, first foggy background signal is removed using a size exclusion to eliminate sources of green fluorescence that were smaller than cells (filter 14.8 µm). After the background signal is removed, the same circle mask as in (A) is applied to mitigate the fluorescence of the well edges.

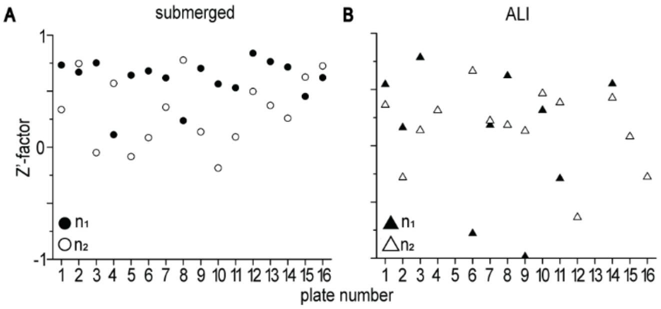

Supplementary Fig. 7. Median Z'- factor for submerged and ALI models.
(A) Median Z'-factor across each plate of 3D bioprinted tissues to measure statistical variance in the submerged model and the **(B)** ALI model. Each plate was completed in duplicate with n1 and n2 shown in closed and open symbols respectively.

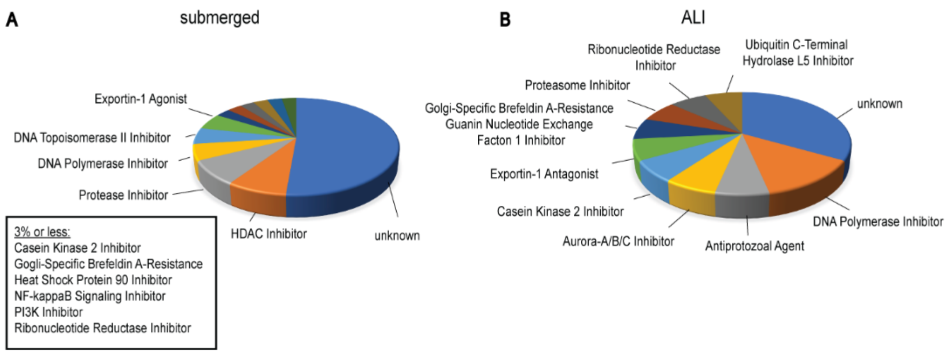

Supplementary Fig. 8. Secondary screen candidate antiviral mechanisms of action.
A proportion of known mechanisms of action for all 106 candidate antivirals in the secondary screen are displayed for submerged models **(A)** and ALI models **(B)**.

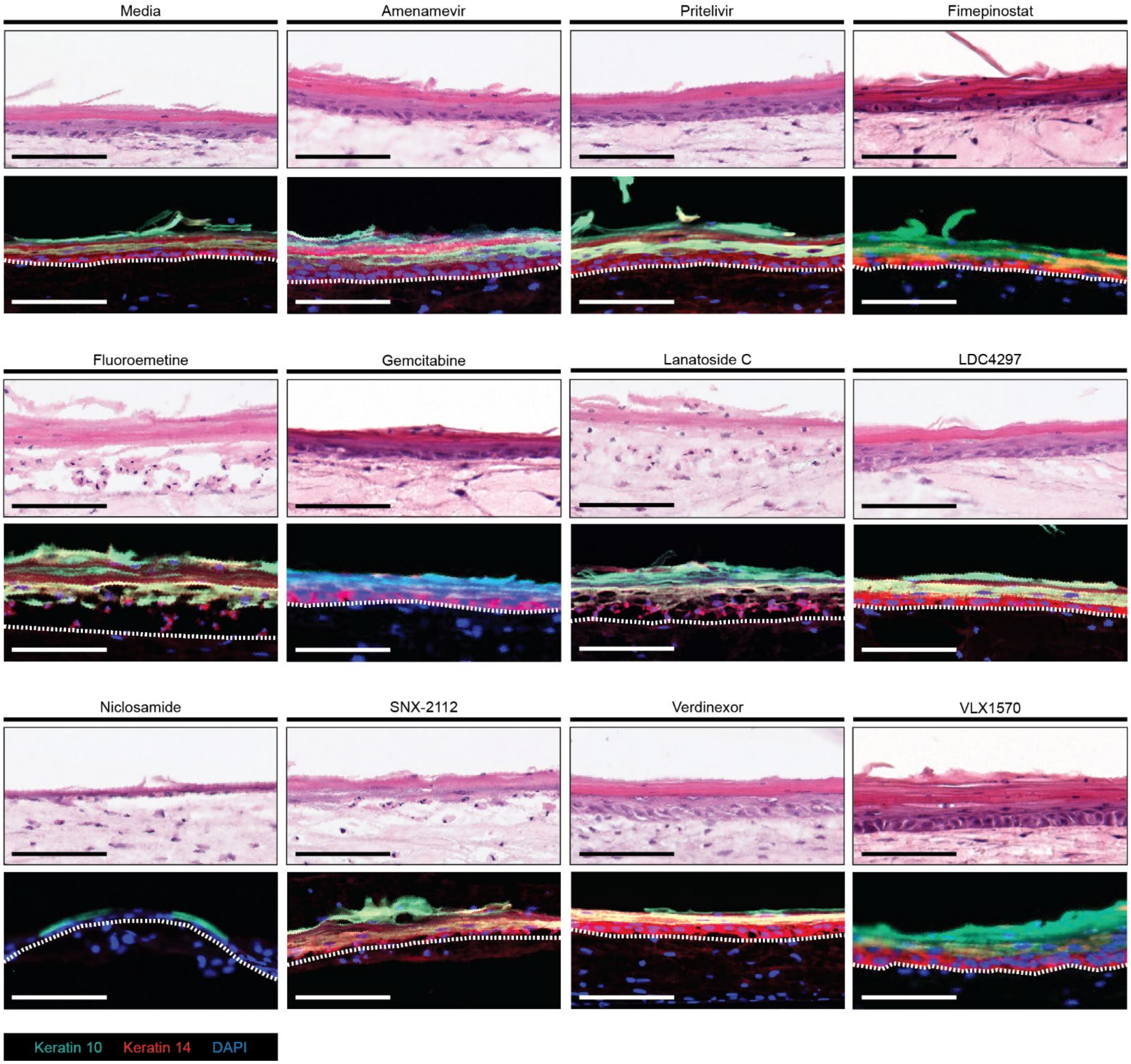

Supplementary Fig. 9. Compound effects on epithelium in 3D bioprinted human skin equivalents. H&E and IHC staining of skin tissues 48 hours after treatment with the top compound candidates at 3.3 μM indicate that most top candidate compounds do not have toxic effects on epithelium development but that FLR, LNT, NCD, and SNX may negatively impact keratinocytes. The dotted white line denotes the basolateral surface of the epidermal equivalent. Scale bar is 100 μm.

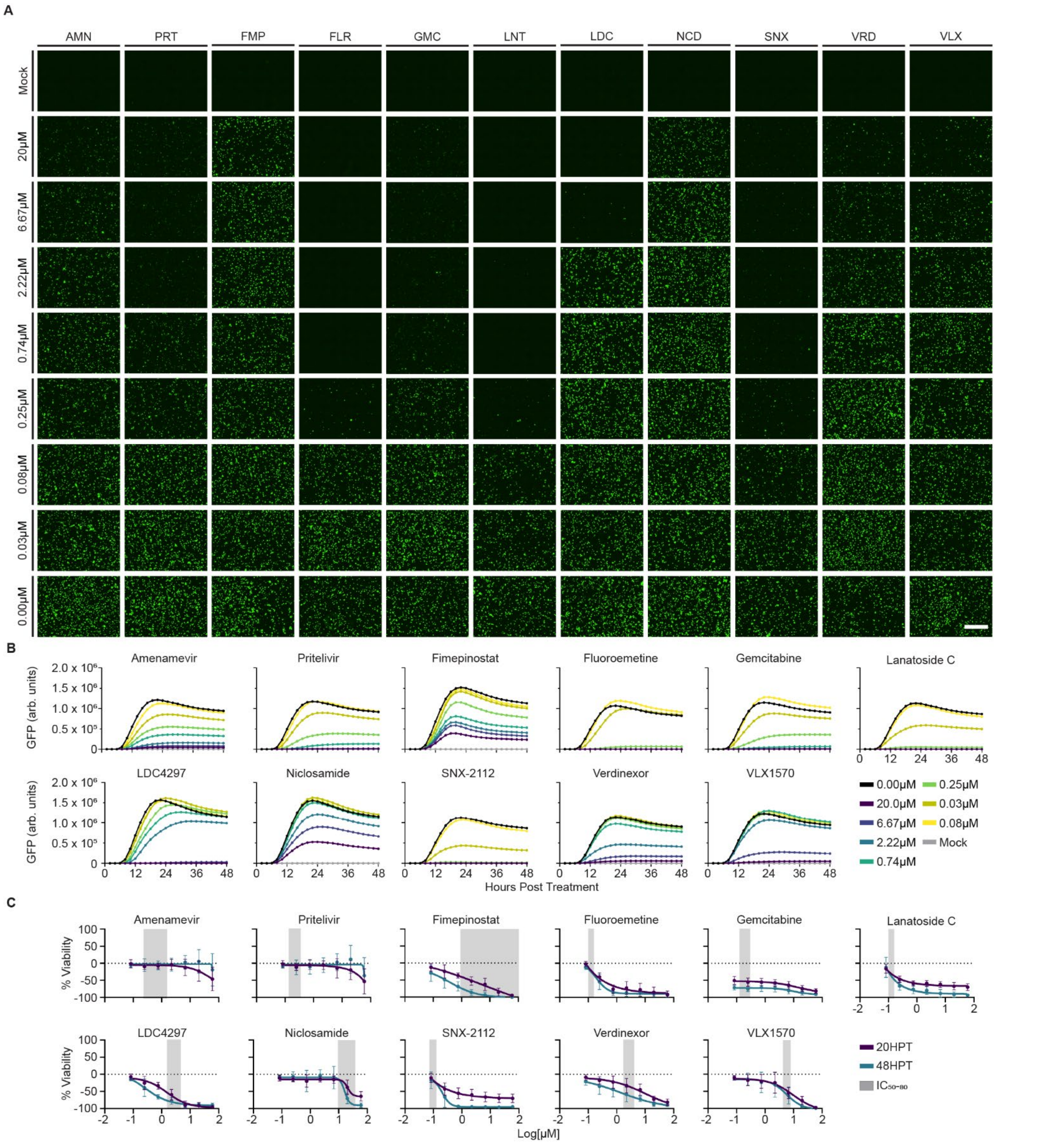

Supplementary Fig. 10. Dose response and cytotoxicity of candidate antivirals in donor-derived keratinocytes.

(A) Live cell images of GFP expression during viral replication at 20HPI. Scale bar is 500 μm. **(B)** Raw GFP integrated fluorescence in infected keratinocytes was calculated from live cell imaging collected every two hours. Each candidate antiviral was analyzed using 1:3 dilutions ranging from 20 μM to 0.08 μM. **(C)** Cytotoxicity of each candidate antiviral in uninfected cells at 20HPT (purple) and 48HPT (blue) compared to keratinocyte average IC_{50} to IC_{80} dose range (grey). All data represent the average of three biological replicates for three donors (N = 9). Error bars represent a single standard deviation.

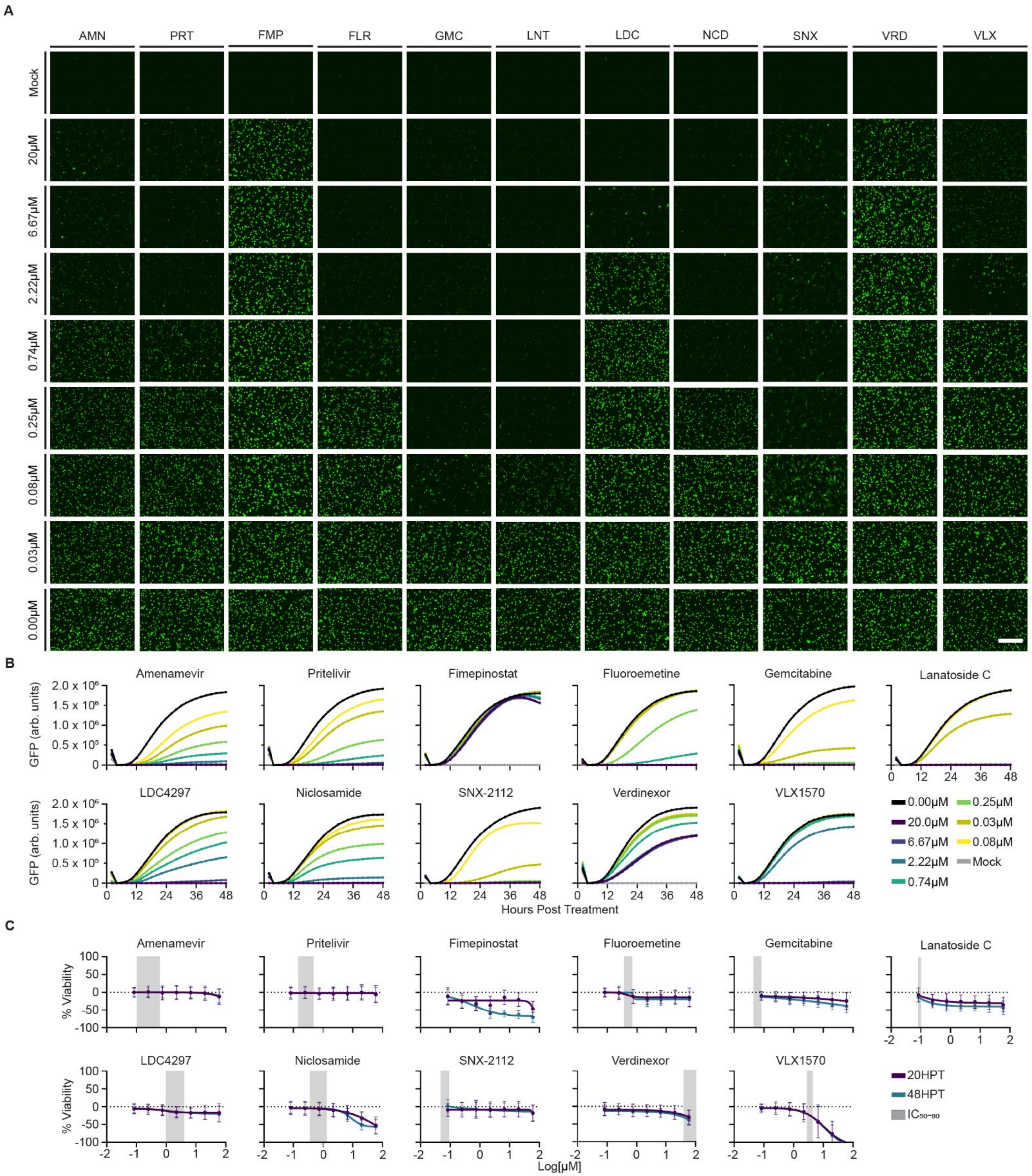

Supplementary Fig. 11. Dose response and cytotoxicity of candidate antivirals in donor-derived fibroblasts.

(A) Representative cell images of GFP expression during viral replication at 20HPI. Scale bar is 500 μm. **(B)** Raw GFP integrated fluorescence in infected fibroblasts was calculated from live-cell imaging collected every two hours. Each candidate antiviral was analyzed using 1:3 dilutions ranging from 20 μM to 0.08 μM. **(C)** Cytotoxicity of each candidate antiviral in uninfected cells at 20HPT (purple) and 48HPT (blue) compared to fibroblast average IC_{50} to IC_{80} dose range (grey). All data represent the average of three biological replicates for three donors (N = 9). Error bars represent a single standard deviation.

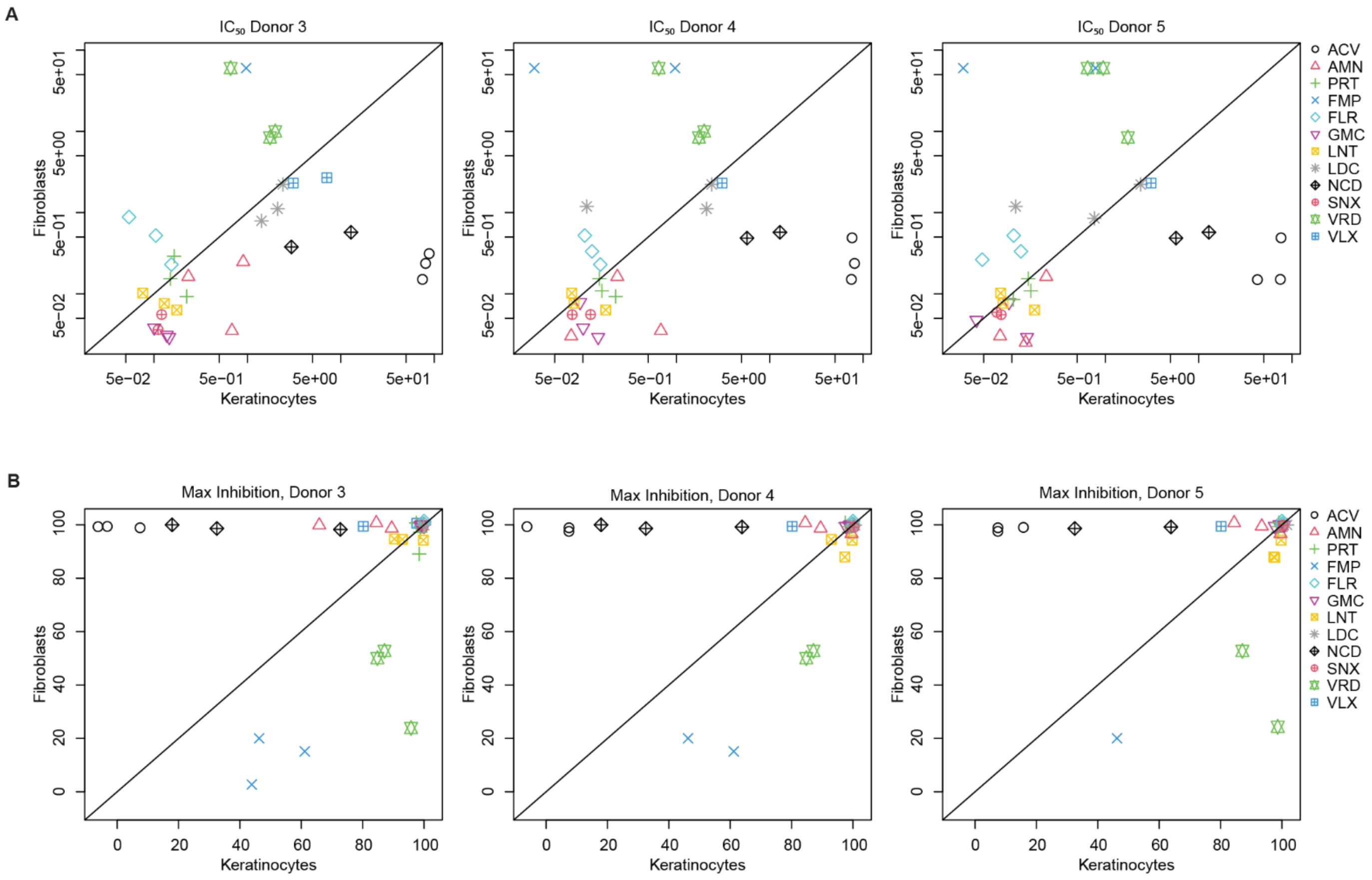

Supplementary Fig. 12. Pairwise comparisons of keratinocytes versus fibroblasts for each individual donor.

(A) Correlation graphs of Log [IC_{50}] values for keratinocytes (X axis) versus fibroblasts (Y axis) for each donor. Symbols represent individual biological replicates (N = 3) **(B)** Correlation graphs of maximum inhibition at 10 μM for each candidate antiviral in keratinocytes (X axis) versus fibroblasts (Y axis) for each donor. Symbols represent individual biological replicates (N = 3).

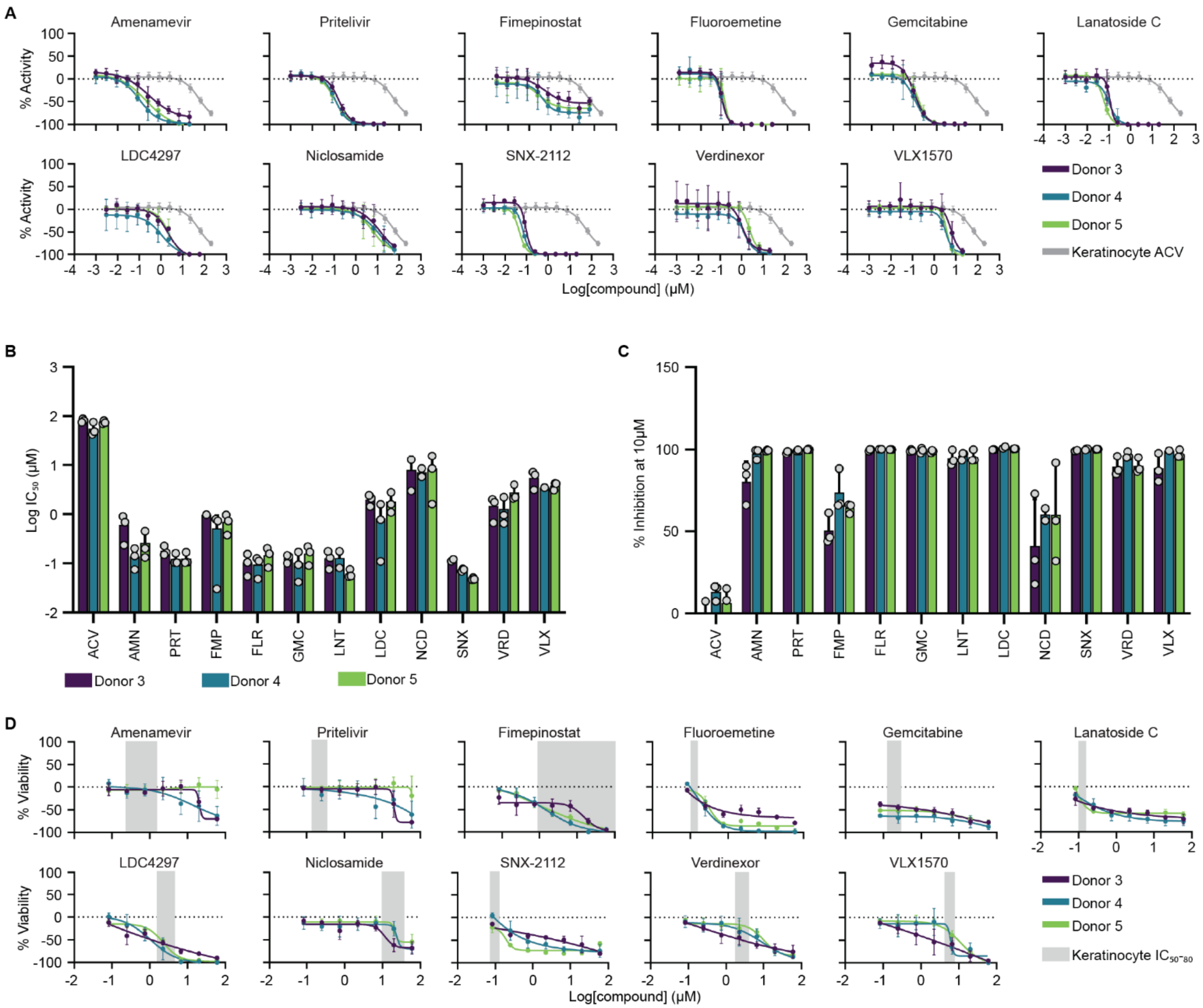

Supplementary Fig. 13. Candidate antiviral potency, efficacy, and cytotoxicity in individual donor-derived keratinocytes.
(A) IC_{50} dose response curves for all twelve top candidate antivirals compared to acyclovir (Donor 3 purple, Donor 4 blue, Donor 5 green, keratinocyte average ACV response grey). **(B)** IC_{50} values for each top candidate antiviral in keratinocytes from Donor 3 (purple), Donor 4 (blue), and Donor 5 (green). **(C)** Total inhibition at 10 μM for each top candidate antiviral is compared among Donor 3 (purple), Donor 4 (blue), and Donor 5 (green). **(D)** CC_{50} dose response curves for all twelve candidate antivirals compared to their respective IC_{50} to IC_{80} dose range. Donor 3 is purple, Donor 4 is blue, Donor 5 is green, and average keratinocyte IC_{50} to IC_{80} dose range is grey. Abbreviations are acyclovir (ACV), amenamevir (AMN), pritelivir (PRT), fimepinostat (FMP), fluoroemetine (FLR), gemcitabine (GMC), lanatoside C (LNT), LDC4297 (LDC), niclosamide (NCD), SNX-2112 (SNX), verdinexor (VRD), and VLX1570 (VLX). All data represent the average of three replicates (N = 3) for each donor. Error bars represent a single standard deviation.

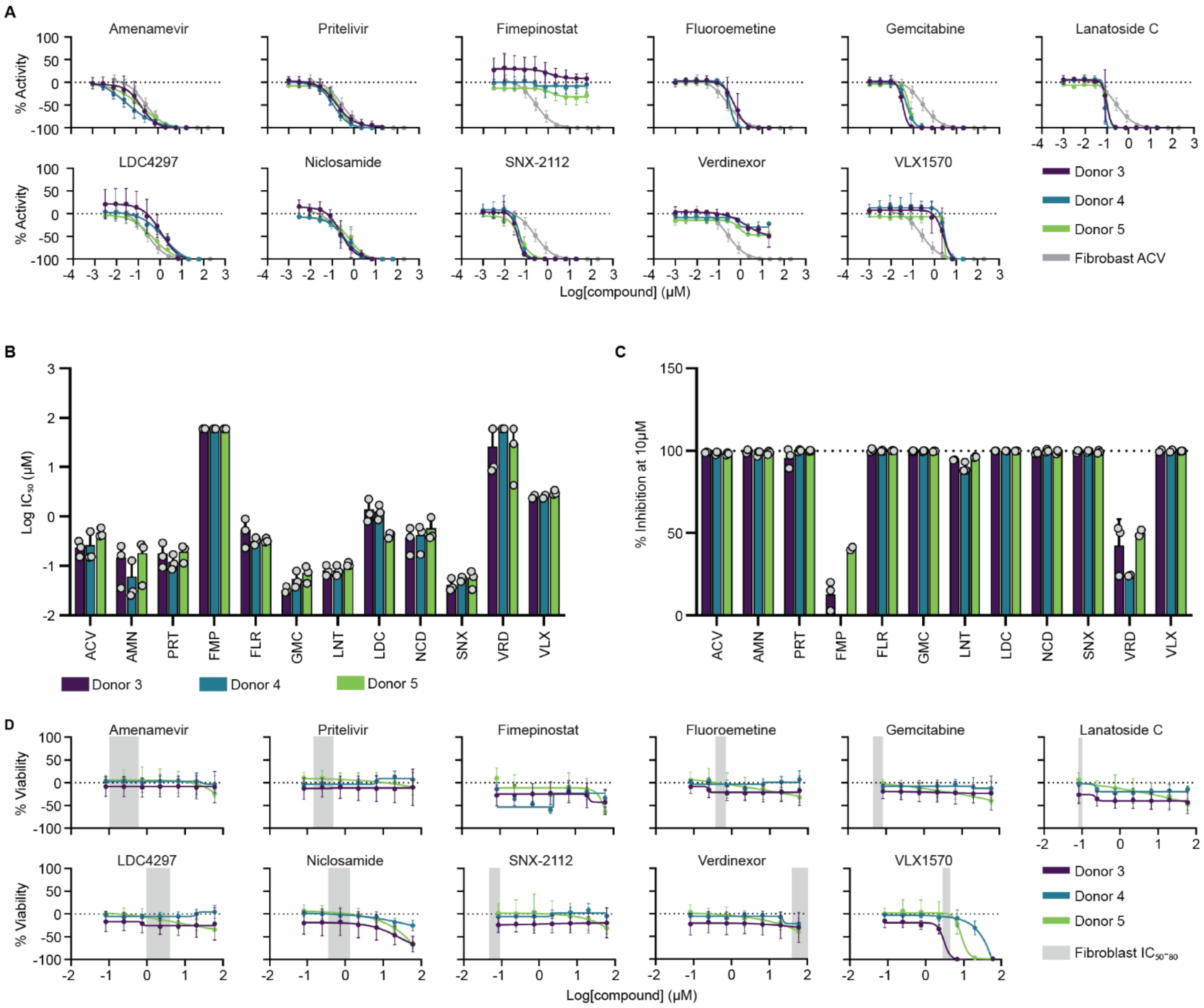

Supplementary Fig. 14. Candidate antiviral potency, efficacy, and cytotoxicity in individual donor-derived fibroblasts.

(A) IC_{50} dose response curves for all twelve top candidate antivirals compared to acyclovir (Donor 3 purple, Donor 4 blue, Donor 5 green, fibroblast average ACV response grey). **(B)** IC_{50} values for each candidate antivirals in fibroblasts from the three independent donors. **(C)** Total inhibition at 10μM for each top candidate antiviral is compared among Donor 3 (purple), Donor 4 (blue), and Donor 5 (green). **(D)** CC_{50} dose-response curves for all twelve candidate antivirals compared to their respective IC_{50} to IC_{80} dose range. Donor 3 is purple, Donor 4 is blue, Donor 5 is green, and the average fibroblast IC_{50} to IC_{80} dose range is grey. Abbreviations are acyclovir (ACV), amenamevir (AMN), pritelivir (PRT), fimepinostat (FMP), fluoroemetine (FLR), gemcitabine (GMC), lanatoside C (LNT), LDC4297 (LDC), niclosamide (NCD), SNX-2112 (SNX), verdinexor (VRD), and VLX1570 (VLX). All data represents average of three replicates (N = 3) for each donor. Error bars represent a single standard deviation.

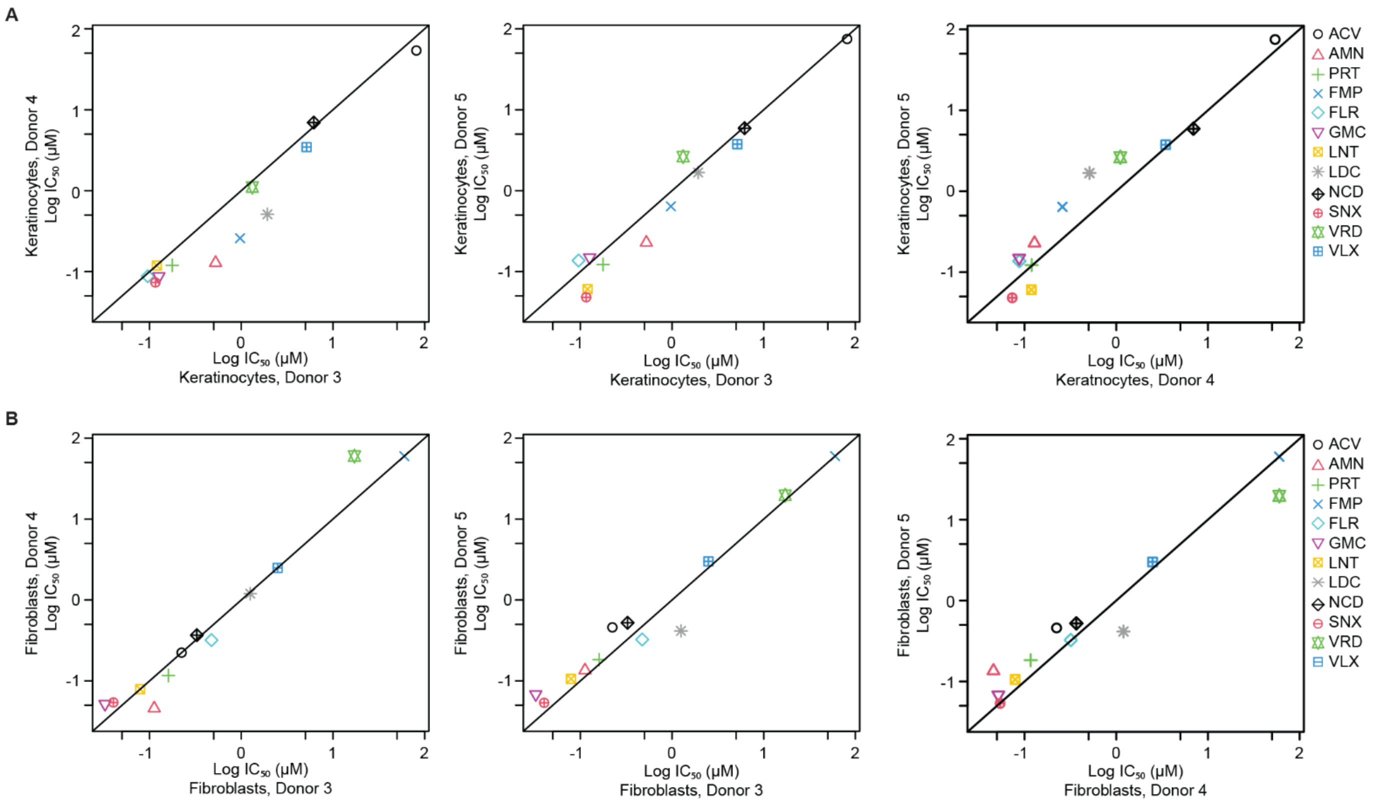

Supplementary Fig. 15. Pairwise comparisons of candidate antiviral IC$_{50}$ values between donors. Correlation graphs of absolute Log [IC$_{50}$] for each candidate antiviral in the specified donors for **(A)** keratinocytes and **(B)** fibroblasts. Symbols represent donor average (N = 3).

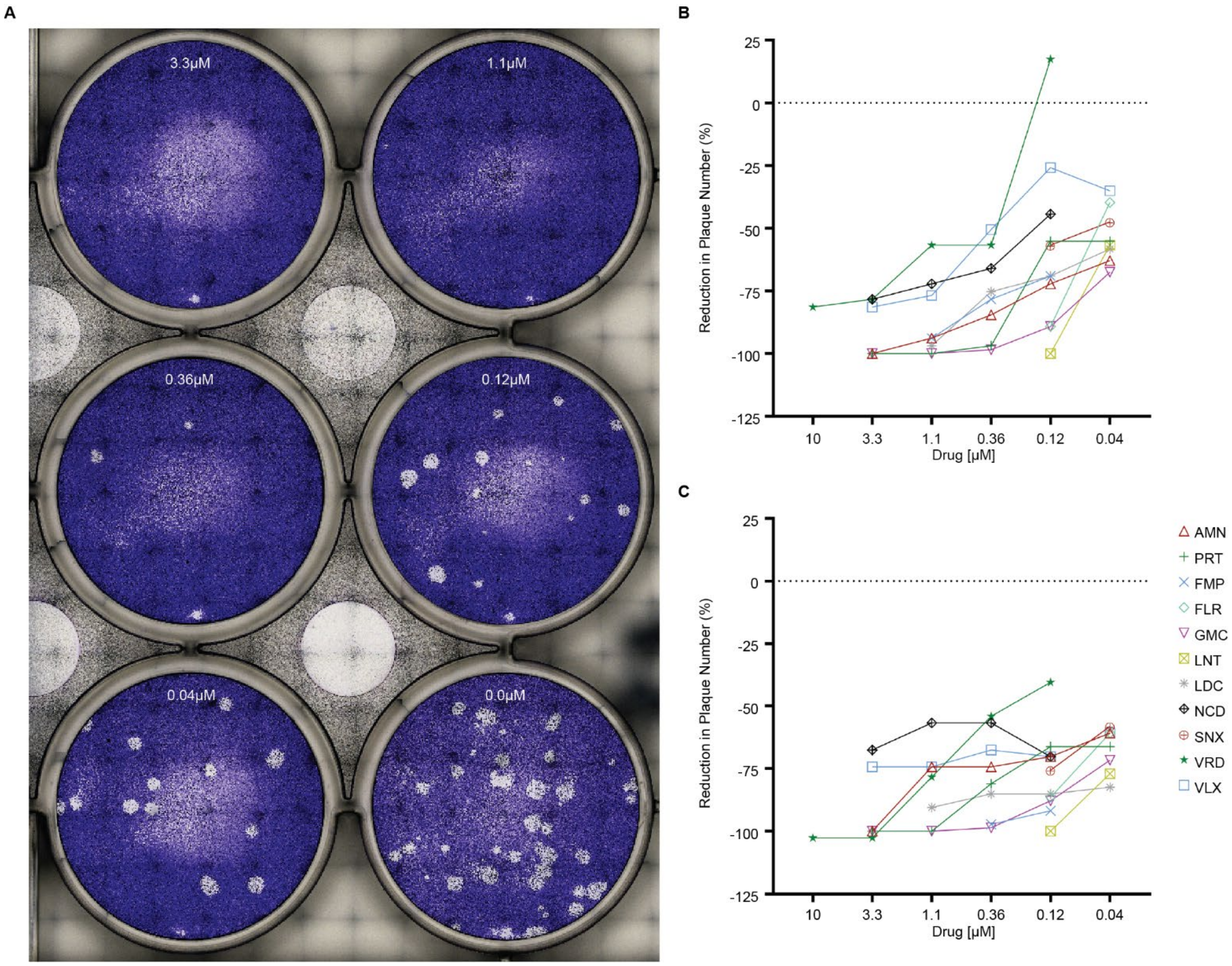

Supplementary Fig. 16. Plaque reduction analysis of candidate antivirals against HSV-1 and HSV-2. Primary keratinocytes were infected at 40 plaque-forming units per well of either HSV-1 K26 or HSV-2 186 with multiple doses of each candidate antiviral. (A) Example plaque reduction assay of pritelivir activity against HSV-2 186. (B&C) Percent of plaque reduction by candidate antivirals against HSV-1 K26 (B) or HSV-2 186 (C). Lines start at the highest dose without visible cytotoxic effects and end at the lowest dose tested for each candidate antiviral. Data represents total plaques counted at each dose (N = 1).

Donor	Age	Sex Assigned at Birth	HSV1 seropositivity	HSV2 seropositivity
1	70s	Female	Positive	Positive
2	60s	Female	Negative	Positive
3	40s	Female	Positive	Positive
4	20s	Male	Negative	Negative
5	20s	Female	Negative	Negative
6	30s	Female	Negative	Negative

Supplementary Table 1. Donor demographic and HSV seropositivity information. Skin punch biopsies were collected from six volunteers. Donor specific keratinocytes and dermal fibroblasts were isolated and matched for comparison.

Candidate antiviral	Literature
Acyclovir	Taylor and Gerriets, Acylcovir, 2023
Adefovir dipivoxil	De Clercq, Clin Microbiol Rev, 2003
Amenamevir	Chono et al, J Antimicrob Chemother, 2010
Bardoxolone methyl	Wyler et al, Nat Commun, 2019
Bortezomib (PS-341)	Schneider et al, mBio, 2019
Cyanein	Ushio et al, Biomed Res, 2009
Cycloheximide	Preston et al, J Gen Virol, 1998
Emetine Dihydrochloride	Andersen et al, Viruses, 2019
Epoxomicin	La Frazia et al, Antivir Ther, 2006
Fiacitabine	Trousdale et al, Invest Ophthalmol Vis Sci, 1981
Ganciclovir	Poole and James, Clin Ther, 2020
Gemcitabine	Denisova et al, J Biol Chem, 2012
GS7340	Tan, Int J Womens Health, 2012
Lanatoside C	Wu et al, Phytomedicine, 2024
MG-132	Ishimaru et al, Sci Rep, 2020
Mitoxantrone	Huang et al, BMC Microbiol, 2019
Niclosamide	Andersen et al, Viruses, 2019
Penciclovir	Poole and James, Clin Ther, 2020
Pritelivir (BAY 57-1293)	Betz et al, Antimicrob Agents Chemother, 2002
SNX-2112	Xiang et al, Bioorg Med Chem Lett, 2012
Trifluorothymidine	Carmine et al, Drugs, 1982
Valaciclovir	Spruance et al, Arch Intern Med, 1996
Valganciclovir HCl	Poole and James, Clin Ther, 2020

Supplementary Table 2. Selected compounds that have published anti-herpes activity.

	IC_{50}	
	Keratinocytes	Fibroblasts
3 versus 4	< 0.001	0.768
3 versus 5	0.214	0.309
4 versus 5	0.024	0.471

Supplementary Table 3: P-value results of pairwise comparisons. Donor-specific differences were calculated using Wald tests based on linear mixed models accounting for the dependent structure of multiple donors with multiple comparison adjustment. Independent biological replicates were completed for each donor. The candidate antiviral potency in both cell types was determined as an absolute IC_{50}.

		Keratinocytes	Fibroblasts
**Amenamevir (AMN)**	IC50 (μM)	0.24	0.10
	CC50 (μM)	> 60.00	> 60.00
	Selectivity	> 250.00	> 600.00
**Pritelivir (PRT)**	IC50 (μM)	0.14	0.15
	CC50 (μM)	55.50	> 60.00
	Selectivity	396.43	> 400.00
**Fimepinostat (FMP)**	IC50 (μM)	0.95	> 60.00
	CC50 (μM)	1.97	1.49
	Selectivity	2.07	< 0.02
**Fluoroemetine (FLR)**	IC50 (μM)	0.10	0.38
	CC50 (μM)	0.31	> 60.00
	Selectivity	3.10	> 157.89
**Gemcitabine (GMC)**	IC50 (μM)	0.13	0.05
	CC50 (μM)	< 0.08	> 60.00
	Selectivity	< 0.62	> 1200.00
**Lanatoside C (LNT)**	IC50 (μM)	0.09	0.08
	CC50 (μM)	0.33	> 60.00
	Selectivity	3.67	> 750.00
**LDC4297 (LDC)**	IC50 (μM)	1.56	0.99
	CC50 (μM)	1.36	> 60.00
	Selectivity	0.87	> 60.00
**Niclosamide (NCD)**	IC50 (μM)	9.90	0.37
	CC50 (μM)	24.47	19.48
	Selectivity	2.47	52.65
**SNX-2112 (SNX)**	IC50 (μM)	0.08	0.05
	CC50 (μM)	0.45	> 60.00
	Selectivity	5.63	> 1200.00
**Verdinexor (VRD)**	IC50 (μM)	1.76	> 60.00
	CC50 (μM)	6.95	> 60.00
	Selectivity	3.95	1.00
**VLX1570 (VLX)**	IC50 (μM)	4.43	3.05
	CC50 (μM)	6.35	8.34
	Selectivity	1.43	2.73

Supplementary Table 4: Comparison of IC50, CC50, and selectivity index for each top candidate antiviral in 2D monoculture. CC_{50} values of > 60 μM indicate that 60 μM doses did not achieve 50% loss of cellular viability. CC_{50} values of < 0.08μM indicate that the lowest dose tested reduced the number of live cells by greater than 50%. IC_{50} and CC_{50} values represent the average of three replicates from each of the three donors (N = 9).

		Submerged	ALI	Keratinocytes	Fibroblasts
Amenamevir (AMN)	IC50 (µM)	0.27	0.15	0.24	0.10
	CC50 (µM)	> 10.00	> 10.00	> 10.00	> 10.00
	Selectivity	> 37.04	> 66.67	> 41.67	> 100.00
Pritelivir (PRT)	IC50 (µM)	0.50	0.19	0.14	0.15
	CC50 (µM)	> 10.00	> 10.00	> 10.00	> 10.00
	Selectivity	> 20.00	> 52.63	> 71.43	> 66.67
Fimepinostat (FMP)	IC50 (µM)	1.45	0.04	0.95	> 10.00
	CC50 (µM)	> 10.00	> 10.00	1.97	1.49
	Selectivity	> 6.90	> 250.00	2.07	< 0.15
Fluoroemetine (FLR)	IC50 (µM)	0.15	0.22	0.10	0.38
	CC50 (µM)	> 10.00	> 10.00	0.31	> 10.00
	Selectivity	> 66.67	> 45.45	3.10	> 26.32
Gemcitabine (GMC)	IC50 (µM)	0.19	0.16	0.13	0.05
	CC50 (µM)	> 10.00	> 10.00	< 0.08	> 10.00
	Selectivity	> 52.63	> 62.50	< 0.62	> 200.00
Lanatoside C (LNT)	IC50 (µM)	0.09	0.08	0.09	0.08
	CC50 (µM)	2.49	> 10.00	0.33	> 10.00
	Selectivity	27.67	> 125.00	3.67	> 125.00
LDC4297 (LDC)	IC50 (µM)	0.67	0.11	1.56	0.99
	CC50 (µM)	> 10.00	> 10.00	1.36	> 10.00
	Selectivity	> 14.93	> 90.91	0.87	> 10.10
Niclosamide (NCD)	IC50 (µM)	0.36	0.11	9.90	0.37
	CC50 (µM)	> 10.00	> 10.00	> 10.00	> 10.00
	Selectivity	> 27.78	> 90.91	1.01	> 27.03
SNX-2112 (SNX)	IC50 (µM)	0.05	0.04	0.08	0.05
	CC50 (µM)	1.14	> 10.00	0.45	> 10.00
	Selectivity	22.80	> 250.00	5.63	> 200.00
Verdinexor (VRD)	IC50 (µM)	0.35	0.15	1.76	> 10.00
	CC50 (µM)	> 10.00	> 10.00	6.95	> 10.00
	Selectivity	> 28.57	> 66.67	3.95	1.00
VLX1570 (VLX)	IC50 (µM)	6.30	0.15	4.43	3.05
	CC50 (µM)	8.41	> 10.00	6.35	8.34
	Selectivity	1.33	> 66.67	1.43	2.73

Supplementary Table 5: Comparison of IC50, CC50, and selectivity index for each top candidate antiviral in 3D and 2D culture. CC_{50} values of > 10 µM indicate that 10 µM doses did not achieve 50% loss of cellular viability. CC_{50} values of < 0.08 µM indicate that the lowest dose tested reduced cellular viability by greater than 50%. IC_{50} values of < 0.04 µM indicate that the lowest does tested resulted in greater than 50% inhibition of virus encoded GFP expression. IC_{50} and CC_{50} values in 3D models represent the average of three replicates (N = 3) while in 2D models represent the average of three replicates from each of the three donors (N = 9).

	Kera v. Fibro	Kera v. Sub	ALI v. Fibro	ALI v. Sub	2D v. 3D
ACV	< 0.001	< 0.001	< 0.001	< 0.001	0.002
AMN	0.032	1.000	1.000	0.602	1.000
PRT	1.000	< 0.001	1.000	0.334	0.004
FMP	< 0.001	0.885	< 0.001	< 0.001	0.033
FLR	< 0.001	1.000	0.235	0.412	1.000
GMC	< 0.001	0.909	0.034	1.000	0.033
LNT	1.000	1.000	1.000	1.000	1.000
LDC	1.000	1.000	0.029	0.008	0.023
NCD	< 0.001	< 0.001	0.001	0.167	0.033
SNX	0.104	1.000	1.000	1.000	0.440
VRD	< 0.001	0.044	< 0.001	1.000	< 0.001
VLX	0.005	0.832	< 0.001	0.004	0.122

Supplementary Table 6: P values comparing the IC50 of each model tested. Candidate antiviral potency was compared between 2D models using Wald tests based on linear mixed models that accounted for the dependent structure of multiple donors. To compare 2D versus 3D, all potency values for each candidate antiviral in keratinocytes (three donors with three biological replicates each) and fibroblasts (three donors with three biological replicates each) were pooled, then compared to all potency values pooled for submerged (N = 3) and ALI (N = 3) models of the same candidate antiviral. 2D was compared to 3D using Wald tests based on mixed models accounting for donor effects. All statistical tests were completed with multiple comparison adjustment.

	Neonatal IC50	Adult IC50
**Submerged Tissues**		
AMN	0.27 ± 0.04	0.38
PRT	0.50 ± 0.23	0.24
FMP	1.48 ± 0.99	0.37
FLR	0.15 ± 0.02	0.13
GMC	0.19 ± 0.08	0.16
LNT	0.09 ± 0.01	0.17
LDC	0.68 ± 0.36	0.70
NCD	0.40 ± 0.49	0.18
SNX	0.06 ± 0.07	0.09
VRD	0.48 ± 0.98	0.52
VLX	6.67 ± 7.17	>10.00
**ALI Tissues**		
AMN	0.16 ± 0.22	0.10
PRT	0.21 ± 0.24	0.25
FMP	< 0.04 ± 0.00	0.24
FLR	0.22 ± 0.14	0.28
GMC	0.17 ± 0.13	0.11
LNT	0.09 ± 0.04	0.14
LDC	0.11 ± 0.04	0.09
NCD	0.11 ± 0.07	0.25
SNX	< 0.04 ± 0.00	< 0.04
VRD	0.17 ± 0.24	0.26
VLX	0.16 ± 0.19	0.24

Supplementary Table 7: Comparison of IC50 values for neonatal versus adult-derived HSEs. 95% confidence intervals were calculated for each candidate antiviral IC50 using three biological replicates in neonatal keratinocyte derived HSEs (N = 3). Candidate antiviral IC50s in adult keratinocyte derived HSEs was determined from a single biological replicate (N = 1).
