# Supplementary material for: Limitations of acyclovir and identification of potent HSV antivirals using 3D bioprinted human skin equivalents
Source: Nat Commun. 2025 Oct 16;16:9200. doi: 10.1038/s41467-025-64245-w (PMC12533247; doi:10.1038/s41467-025-64245-w)
Supplement: Supplementary file 2 — Description of Additional Supplementary Files [file 41467_2025_64245_MOESM2_ESM.pdf]

Title: Supplementary Data 1.

Description: A library of 738 compounds and their mechanisms of action used in the anti-HSV screening studies.

Title: Supplementary Data 2.

Description: Maximum responses of antiviral effect on HSV-1 infectivity (GFP) and fibroblast viability (tdTomato) for each of the two repeats in the primary screen in the submerged and ALI models.

Title: Supplementary Data 3.

Description: Concentration-response curve class (CC-v2), efficacy, maximum response, and IC50 of the 106 and 41selected compounds in the submerged and ALI models.

Title: Supplementary Data 4.

Description: Concentration-response curve class (CC-v2), efficacy, maximum response, and IC50 of the top 11 compounds in the submerged and ALI models in triplicate.

Title: Supplementary Data 5.

Description: Concentration-response curve class (CC-v2), efficacy, maximum response, and IC50 of the top 11 compounds in the submerged and ALI models using donor-derived keratinocytes
