# Supplementary material for: Limitations of acyclovir and identification of potent HSV antivirals using 3D bioprinted human skin equivalents
Source: Nat Commun. 2025 Oct 16;16:9200. doi: 10.1038/s41467-025-64245-w (PMC12533247; doi:10.1038/s41467-025-64245-w)
Supplement: Supplementary file 8 — Reporting Summary [file 41467_2025_64245_MOESM8_ESM.pdf]

nature portfolio

Corresponding author(s): Jia Zhu & Marc Ferfer

Last updated by author(s): Sep 10, 2025

Reporting Summary

Nature Portfolio wishes to improve the reproducibility of the work that we publish. This form provides structure for consistency and transparency in reporting. For further information on Nature Portfolio policies, see our Editorial Policies and the Editorial Policy Checklist.

Statistics

For all statistical analyses, confirm that the following items are present in the figure legend, table legend, main text, or Methods section.

n/a	Confirmed	
☐	☒	The exact sample size (n) for each experimental group/condition, given as a discrete number and unit of measurement
☐	☒	A statement on whether measurements were taken from distinct samples or whether the same sample was measured repeatedly
☐	☒	The statistical test(s) used AND whether they are one- or two-sided<br>*Only common tests should be described solely by name; describe more complex techniques in the Methods section.*
☐	☒	A description of all covariates tested
☐	☒	A description of any assumptions or corrections, such as tests of normality and adjustment for multiple comparisons
☐	☒	A full description of the statistical parameters including central tendency (e.g. means) or other basic estimates (e.g. regression coefficient) AND variation (e.g. standard deviation) or associated estimates of uncertainty (e.g. confidence intervals)
☐	☒	For null hypothesis testing, the test statistic (e.g. F, t, r) with confidence intervals, effect sizes, degrees of freedom and P value noted<br>*Give P values as exact values whenever suitable.*
☒	☐	For Bayesian analysis, information on the choice of priors and Markov chain Monte Carlo settings
☒	☐	For hierarchical and complex designs, identification of the appropriate level for tests and full reporting of outcomes
☒	☐	Estimates of effect sizes (e.g. Cohen's d, Pearson's r), indicating how they were calculated

Our web collection on statistics for biologists contains articles on many of the points above.

Software and code

Policy information about availability of computer code

Data collection	3D image capture: MetaXpress Moldev Microscope<br>2D image capture: Sartorius IncuCyte S3<br>Plaque Reduction Assay image capture: Nikon Eclipse Ti2
Data analysis	3D image data analysis: MetaXpress Analysis Software<br>2D image data analysis: IncuCyte Analysis Software 2023A<br>Plaque Reduction image stitching and analysis: NIS Elements 5.21.03<br>Graphing and plotting: Graphpad Prism 9<br>Statistical analysis: R (R core team) with linear mixed model lme4 package

For manuscripts utilizing custom algorithms or software that are central to the research but not yet described in published literature, software must be made available to editors and reviewers. We strongly encourage code deposition in a community repository (e.g. GitHub). See the Nature Portfolio guidelines for submitting code & software for further information.

Data

Policy information about availability of data

All manuscripts must include a data availability statement. This statement should provide the following information, where applicable:

- Accession codes, unique identifiers, or web links for publicly available datasets
- A description of any restrictions on data availability
- For clinical datasets or third party data, please ensure that the statement adheres to our policy

All data supporting the findings of this study are available within the paper and its Supplementary Information. Analysis code is available from the Github repository in https://github.com//youyifong/drug_discovery_HSV/.

Research involving human participants, their data, or biological material

Policy information about studies with human participants or human data. See also policy information about sex, gender (identity/presentation), and sexual orientation and race, ethnicity and racism.

Reporting on sex and gender	Primary keratinocytes and fibroblasts were collected from skin of both female (n=5) and male (n=1) donors recruited by the Virology Research Clinic at the University of Washington. Donor-specific keratinocyte and dermal fibroblast cells were matched for comparison. Due to the limited number of donors, sex and/or gender were not taken into account during data analysis.
Reporting on race, ethnicity, or other socially relevant groupings	All primary cell donors were white; two of the six primary donors were of hispanic or latino ethnicity. Our data analysis does not consider race or ethnicity during analysis due to the small donor number. We have moved this data from Supplemental Table 1, as this has the risk of identifying individual participants.
Population characteristics	Primary donor sex, age shown in decades, and HSV1/2 seropositivity information is provided in Supplemental Table 1. Biological sex was biased towards females as herpes simplex more commonly presents in biological females.
Recruitment	Advertisements for the study were posted in local newspapers throughout the Seattle area. This might result in self-selecting for individuals who read those news sources and are local to the area.
Ethics oversight	The study protocol involving human specimens was approved by the University of Washington Institutional Review Board Committee (STUDY00004312), and written informed consents were obtained from all participants.

Note that full information on the approval of the study protocol must also be provided in the manuscript.

Field-specific reporting

Please select the one below that is the best fit for your research. If you are not sure, read the appropriate sections before making your selection.

☒ Life sciences ☐ Behavioural & social sciences ☐ Ecological, evolutionary & environmental sciences

For a reference copy of the document with all sections, see nature.com/documents/nr-reporting-summary-flat.pdf

Life sciences study design

All studies must disclose on these points even when the disclosure is negative.

Sample size	We implemented a screen of 738 compounds with broad targets and a wide range of mechanisms of action utilizing our 3D bioprinted HSE assay platform. To determine if our assay is amenable to high-throughput screening (HTS), we calculated the median Z'-factor, a measure of the robustness of the assay. A score that is Z'>0.5 denotes a robust assay window for screening, 0.5>Z'>0 indicates a marginal assay window and that the screen needs replicates, and a Z'<0 means that the assay window is not robust enough for screening. In the submerged model, the Z' was 0.57, and for the ALI model, the Z' was 0.13, indicating that the submerged assay is highly robust while the ALI assay is moderately robust, demonstrating that our 3D bioprinted assay platform was amenable to HTS. Initial candidate antiviral screens of 738 compounds were performed for reproducibility in two independent replicates as a selection criteria for further evaluation. The top 11 candidate compounds were studied using at least three independent experiments in both 3D and 2D models for statistical analysis.
Data exclusions	No data excluded in 3D data. Images with non-biological autofluorescent debris were excluded from 2D IncuCyte data analysis.
Replication	All experiments were independently replicated using distinct biological replicates as indicated in the figure legends.
Randomization	Experimental groups were determined by cell type or model type (e.g. 3D vs 2D). Mock or candidate drug treatments were applied using a preset plate layout and were not selectively applied to 3D tissues or 2D monoculture wells based on any other characteristics.
Blinding	Drug treatment was blinded in large scale screen Investigators were blinded to compounds during data collection and analysis for the primary and secondary screens.

Reporting for specific materials, systems and methods

We require information from authors about some types of materials, experimental systems and methods used in many studies. Here, indicate whether each material, system or method listed is relevant to your study. If you are not sure if a list item applies to your research, read the appropriate section before selecting a response.

Materials & experimental systems

n/a	Involved in the study
☐	☒ Antibodies
☐	☒ Eukaryotic cell lines
☒	☐ Palaeontology and archaeology
☒	☐ Animals and other organisms
☒	☐ Clinical data
☒	☐ Dual use research of concern
☒	☐ Plants

Methods

n/a	Involved in the study
☒	☐ ChIP-seq
☒	☐ Flow cytometry
☒	☐ MRI-based neuroimaging

Antibodies

Antibodies used

Primaries:
rabbit anti-human loricrin: (1:1,500, ABCAM, ab198994, lot 1063759-10)
rabbit anti-human filaggrin: (1:5,000, ABCAM, ab221155, lots 1029031-2, GR3456492-1, and GR3380631-6)
rabbit anti-human Keratin 10: (1:2,000, ABCAM, ab234313, lots 1054991-1 and 1069014-1)
rabbit anti-human Keratin 14: (1:500, ABCAM, ab119695, lot 1001149-31)

Secondaries:
Akoya Biosciences Opal 520 Reagent Pack: (1:150, Fisher Sci, NC1601877)
Akoya Biosciences Opal 690 Reagent Pack: (1:150, Fisher Sci, NC1605064)

Validation

All antibodies are commercially available and validated by the manufacturer, and found to display expected staining patterns in our staining applications.
Abcam: Antibody specificity is confirmed by looking at cells that either do or do not express the target protein within the same tissue. Initially, our scientists will review the available literature to determine the best cell lines and tissues to use for validation. We then check the protein expression by IHC/ICC to see if it has the expected cellular localization (Figure 3). If the localization of the signal is as expected, this antibody will pass and is considered suitable for use in IHC/ICC.

Eukaryotic cell lines

Policy information about cell lines and Sex and Gender in Research

Cell line source(s)

Neonatal human dermal fibroblasts (HDFN, Zen Bio DFN-F), Neonatal Normal Human Epithelial Keratinocytes (NHEKN, ScienCell 2100), . Vero cells were originally sourced from ATCC (CCL-81).

Authentication

Primary cells were isolated as described in the materials and methods and characterized by morphology and growth conditions. Cells were not authenticated in house.

Mycoplasma contamination

Cell lines were tested for mycoplasma and presented as negative.

Commonly misidentified lines
(See ICLAC register)

No commonly misidentified cell lines were used in this study. Primary cell types were confirmed through visual inspection of cellular morphology and the use of cell-type specific culture conditions.

Plants

Seed stocks

Report on the source of all seed stocks or other plant material used. If applicable, state the seed stock centre and catalogue number. If plant specimens were collected from the field, describe the collection location, date and sampling procedures.

Novel plant genotypes

Describe the methods by which all novel plant genotypes were produced. This includes those generated by transgenic approaches, gene editing, chemical/radiation-based mutagenesis and hybridization. For transgenic lines, describe the transformation method, the number of independent lines analyzed and the generation upon which experiments were performed. For gene-edited lines, describe the editor used, the endogenous sequence targeted for editing, the targeting guide RNA sequence (if applicable) and how the editor was applied.

Authentication

Describe any authentication procedures for each seed stock used or novel genotype generated. Describe any experiments used to assess the effect of a mutation and, where applicable, how potential secondary effects (e.g. second site T-DNA insertions, mosiacism, off-target gene editing) were examined.
